# Privacy preserving data visualizations

**DOI:** 10.1140/epjds/s13688-020-00257-4

**Published:** 2021-01-07

**Authors:** Demetris Avraam, Rebecca Wilson, Oliver Butters, Thomas Burton, Christos Nicolaides, Elinor Jones, Andy Boyd, Paul Burton

**Affiliations:** 1 Population Health Sciences Institute, Newcastle University, Newcastle Upon Tyne, UK; 2 Department of Business and Public Administration, University of Cyprus, Nicosia, Cyprus; 3 Department of Public Health, Policy and Systems, Institute of Population Health, University of Liverpool, Liverpool, UK; 4 Department of Computer Science, University of Oxford, Oxford, UK; 5 Nireas Research Center, University of Cyprus, Nicosia, Cyprus; 6 Sloan School of Management, Massachusetts Institute of Technology, Massachusetts, USA; 7 Department of Statistical Science, University College London, London, UK; 8 Population Health Sciences, Bristol Medical School, University of Bristol, Bristol, UK

**Keywords:** Sensitive data, Data visualizations, Disclosure control, Privacy protection, Anonymization

## Abstract

Data visualizations are a valuable tool used during both statistical analysis and the interpretation of results as they graphically reveal useful information about the structure, properties and relationships between variables, which may otherwise be concealed in tabulated data. In disciplines like medicine and the social sciences, where collected data include sensitive information about study participants, the sharing and publication of individual-level records is controlled by data protection laws and ethico-legal norms. Thus, as data visualizations – such as graphs and plots – may be linked to other released information and used to identify study participants and their personal attributes, their creation is often prohibited by the terms of data use. These restrictions are enforced to reduce the risk of breaching data subject confidentiality, however they limit analysts from displaying useful descriptive plots for their research features and findings.

Here we propose the use of anonymization techniques to generate privacy-preserving visualizations that retain the statistical properties of the underlying data while still adhering to strict data disclosure rules. We demonstrate the use of (i) the well-known *k*-anonymization process which preserves privacy by reducing the granularity of the data using suppression and generalization, (ii) a novel deterministic approach that replaces individual-level observations with the centroids of each *k* nearest neighbours, and (iii) a probabilistic procedure that perturbs individual attributes with the addition of random stochastic noise. We apply the proposed methods to generate privacy-preserving data visualizations for exploratory data analysis and inferential regression plot diagnostics, and we discuss their strengths and limitations.

## Introduction

Formal statistical techniques based on quantitative inferences are crucial and centrally involved in every data-driven program of scientific research. However, data analysis without the generation of informative data visualizations often does not help the analysts and readers, especially those without strong statistical backgrounds, to understand complex research questions, nuanced relationships between variables and statistical outputs. Data visualizations are therefore valuable tools that can be used during exploratory and inferential analysis, the subsequent derivation and interpretation of conclusions, and the publication of the results and findings [1–3]. Informative graphs along with conventional statistical analysis give meaning to the otherwise meaningless numbers in datasets, by detecting the statistical properties and attributes of the data, displaying associations and their magnitudes between different variables and visually inferring study-level statistical hypotheses.

The importance of graphical representations when exploring an underlying dataset is illustrated by the fact that different datasets can feature identical summary statistics (e.g. mean, standard deviation, Pearson’s correlation, etc.) but the spatial distribution of the data displayed in a real coordinate space (e.g. in a 2-dimensional scatter plot) might vary substantially [4]. This means that the underlying structure of the data remains hidden, if informative graphs are not created at the analysis stage. In addition, plots can enhance the clarity of data presentation and can help readers to understand the research findings and to derive clear and correct conclusions. In particular, there is strong evidence that the human brain processes visualized data more quickly and more efficiently than other data representations [5, 6].

The power of displaying underlying patterns and relationships using data visualizations provides an important contribution to science in general [7]. However, in disciplines like medicine and social sciences, where collected data include sensitive information about the data subjects (e.g. study participants, patients in a hospital, cohort members, etc.), this advantage is constrained by the privacy-related restrictions. Sensitive information is protected by data protection laws – like the *EU General Data Protection Regulation* (GDPR) [8] – and ethico-legal norms – such as the *Common Law duty of Confidentiality* in the UK – which control how Personal Data are shared, accessed, processed and published. These protections should also be applied, therefore, to graphical representations of data, as published plots, without any processing, can provide as much information as publishing the raw data. Even when the graphs visualize data from rich datasets, containing large volumes of data items on many data subjects, and even if the data are pre-processed to be pseudonymized, some individuals might still be identifiable due to unique combinations of their attributes in the dataset. For this reason, under GDPR legislation, pseudonymized data are still considered identifiable Personal Data [9]. This can be particularly problematic, as standard graphical tools do not consider privacy risk and therefore most commonly generated plots may result in the breach of study participants’ sensitive information.

*Potentially-disclosive* visualizations may be described as graphical methods allowing sensitive information to be inferred or deduced, either directly or by combining multiple representations. This may in some circumstances risk disclosure of identity, particularly based on outlying observations. Once identity is disclosed, there is inevitably a risk that new information, not in the public domain, may be inferred about that individual. In addition, there is a risk of deductive attacks on the privacy of other people included in a dataset, if an attacker progressively excludes identified individuals. A characteristic example of a potentially-disclosive visualization is the conventional scatter plot, which directly exposes the exact values of two attributes for every individual in a dataset. When one of the two variables in a scatter plot is a direct identifier (e.g. an ID indicator, the exact date of birth, etc.) there is direct disclosure of the second variable for each individual. When both of the variables are not direct identifiers, an individual may still be identified (with his/her two attributes being disclosed) especially for outlying and unique observations. For graphs where data are aggregated in bins (e.g. histograms) or grids (e.g. heat maps) the identification of individuals is harder, based on the level of aggregation. However, it is often unavoidable for outlying observations and in the absence of disclosure controls. On the other hand, the use of an increased level of aggregation can be a drawback, as there is a great chance to lose important information (e.g. the distribution displayed in a histogram with wide bins, might considerably differ from the actual distribution of the variable).

To give an example, let us assume that we have a database of medical records from a specific hospital, like the example shown in Table 1. The dataset includes the full name of each patient (direct identifier), the age of each person in years (potentially direct identifier especially for unique values), their blood glucose level (non-identifier, sensitive information), and a binary indicator where ‘Yes’ or ‘No’ denotes whether a person has diabetes or not (non-identifier, sensitive information). If we generate a scatter plot between age and glucose level, and if we know that Alice is a patient in the hospital and her age is 25, then we can directly disclose her blood glucose level, which is sensitive personal information and also an indicator for diabetes. If the hospital intends to directly release a statistical report on the age distribution of diabetic patients in the database, representing that in a histogram, then private information of individuals may be also leaked. For example, if we know that Alice is the only person aged between 20 and 30 years in the hospital, and if the bins of the histogram represent single years of age or even if they represent 10-year intervals with breaks at each decade, then we can confirm that Alice has diabetes.

**Table 1:** Exemplar sensitive data

Name	Age	Glucose	Diabetes
Alice Smith	25	237	Yes
Bob Taylor	34	186	Yes
Frank Jones	33	165	Yes
Ivy Smith	42	80	No
Jim Davies	12	190	Yes
…	…	…	…

Despite these disclosure risks, it is clear that with appropriate statistical disclosure controls [10] it may be possible to generate privacy-protected graphical outputs. *Privacy-preserving* visualizations can therefore be defined as graphs that control the risk of disclosure by applying permutations to the real data points. Of course, there are cases where an attacker might have enough external information to identify other information from “processed” graphs, however the realistic objective of privacy-preserving techniques may be viewed as making such disclosure attempts not reasonable likely, and easier to detect via contemporaneous or *post hoc* data processing. The risk of disclosure based on graphical outputs of sensitive data is often mitigated by restrictions placed in the data use terms and conditions. For example, many Data Safe Haven and Trusted Research Environments [11, 12] specify that no research outcomes, including plots, are allowed to be published, or sometimes even created, without risk evaluation and human checking of disclosure risks in that output. But, this is a time consuming and error-prone process which has a substantive potential to miss things. So there is a resource issue (cost to research), a time issue (delays for the researcher) and a risk/reputation issue (failure to detect disclosive content which then brings the data science into disrepute and threatens the on-going availability of data). On the other hand, the creation of privacy-preserving graphics (and other statistical outputs) might ideally be managed at the analysis stage, thus circumventing the concern that a block any earlier in data-processing runs the risk of degrading the quality of the data, whilst at any later stage, runs the risk of requiring a repetition of the analysis with stricter disclosure controls (thus creating time delays).

The consideration and control of disclosure risk is best approached through risk based assessments. Once risk is identified, then tools and techniques are needed to mitigate these. In this paper, we demonstrate how statistical techniques of data anonymization can be used for the development of privacy-preserving visualizations. Here we use the following techniques:

(i) *k*-*anonymization*: uses a combination of suppression and generalization of individual attributes until each row in a given dataset becomes identical to at least $k-1$ other rows [13]. *Suppression* is achieved when certain values of attributes with high risk of identification (usually unique extreme values or outliers) are replaced by arbitrary symbols, or missing value indicators, or are deleted completely from the dataset. Under *generalization*, individual values are replaced with broader categories (e.g. a vector indicating the continuous age in years can be replaced by a vector of wider age groups).
(ii) *deterministic anonymization*: for each individual attribute we generate a cluster with its $k-1$ nearest by distance neighbours and then replace its value by the average (geocentroid) of the attributes in the cluster. This method differs from micro-aggregation [14], as one distinct cluster is formed for each single data record.
(iii) *probabilistic anonymization*: perturbs the individual values of a variable with the addition of random stochastic noise of variance equal to a proportion of the actual dispersion of the underlying variable [15].

This paper is structured as follows: In the ‘Methods’ section we describe the three anonymization techniques used in generating privacy-preserving graphs (Sects. 2.1–2.3). In the ‘Data’ section, we present the simulated data used for the graphical illustrations of this study. In the ‘Results’ section we first demonstrate the privacy-preserving graphical displays for exploratory data analysis (Sect. 4.1) and for regression plot diagnostics (Sect. 4.2), and we then present the performance of the used techniques with variation of their parameters (Sects. 4.3–4.5) and on different sample sizes (Sect. 4.6). Finally, in the ‘Discussion’ section we discuss the findings of our study and illustrate the strengths and limitations of the methods used.

## Methods

### *k*-anonymization

The *k*-anonymization method is a commonly used data protection technique [13, 16, 17]. It converts a set of individual-level data into a *k*-anonymous form, which means that the combined information for each individual in a given dataset cannot be distinguished from the combined information of at least $k-1$ other individuals in the same dataset. To achieve *k*-anonymous data, the individual attributes are suppressed or generalized, until each row in the dataset becomes identical with at least $k-1$ other rows. Using suppression, certain values of attributes with high risk of identification (usually unique indicators, extreme values or outliers) are deleted or replaced by meaningless symbols. Using generalization, granular values of attributes are replaced with broader categories or intervals. However, it is difficult, if not impossible, to select an optimal method/protocol that produces *k*-anonymous data [18, 19]. For each data setting, the preliminary data processors must therefore determine how many times to apply suppression and/or generalization and in which combination, in order to achieve the value of *k*-anonymity required.

Suppression is also used to protect sensitive information in tabular summary statistics. A characteristic example is the application of suppression of cells in contingency tables, where counts of categorical variables or quantitative measures of continuous variables (e.g. the mean) are tabulated across a number of factors. Known as the *small cell counts suppression rule*, the method specifies a threshold indicating the minimum non-zero value of units allowed in any of the cells in the contingency table, and if any cell has less counts than this threshold, its value is removed from the table [20]. If only one cell requires cell suppression, then at least one other complementary cell must be suppressed, in order to avoid calculation of the suppressed values from the marginal totals [21]. In special situations null cells (cells with zero counts), or 100% cells (cells where all contributors within a category have a particular characteristic) are considered sensitive and must also be protected from identification risks.

Generalization can be applied both to quantitative variables (e.g. combining continuous ages given in years into broader categories of age-groups) and for qualitative variables (e.g. combining locations given as street names into names for wider areas). One disclosure limitation method that can be used to generalize individual-level attributes is micro-aggregation [14]. Micro-aggregated data are obtained when individual values of variables are clustered in groups of size of at least *k* and each individual value is replaced by the average value over each group. The groups are determined by criteria of maximal similarity – such as the proximity of the data values.

Here, we use both components of *k*-anonymization – suppression and generalization – to generate privacy-preserving visualizations of data aggregated in bins or cells. Such plots include the histogram which is a discretized representation of the probability distribution of a numerical variable, and the heat map which displays the density distribution of two variables tabulated in grids. The protection of sensitive information in these outputs, is achieved through suppression of cells, grids or bins with *k* or less counts, or generalization of the variables into broader intervals. For the suppression rule we use the threshold value of $k=3$ as a baseline value and we vary this parameter in Sect. 4.3 to explore its effect on the data visualizations. We note here, that the *k*-anonymization method cannot be applied to plots of ungrouped data (e.g. scatter plots), however we micro-aggregate the data and apply the method to demonstrate its poor performance on such plots.

### Deterministic anonymization

Deterministic anonymization is a novel method, being proposed here, that provides a quite different approach to generating privacy-preserving visualizations. Our proposal is based on the use of the *k-Nearest Neighbours*, a commonly used algorithm in machine learning and data mining applications [22, 23]. Based on this, the deterministic anonymization of an underlying dataset can be achieved through the following procedure: we first standardize each continuous variable using z-score transformations such that each variable is centralized to zero mean and scaled to unit variance. With z-score transformations, the mean of a given variable is subtracted from each individual value and each difference is divided by the variable’s standard deviation. We then find the $k-1$ nearest neighbours of each individual data point; the distance between data points being determined by the Euclidean distance metric. Alternatively, the Mahalanobis distance metric [24] can be used without a requirement for initial standardization. This is because the Mahalanobis distance accounts for the scale and the variance of each variable and all pairwise bivariate covariances. In a two-dimensional space, the Mahalanobis distance, Δ, between two vectors ***x*** and ***y*** is given mathematically by $\Delta =\sqrt{(\boldsymbol{x}-\boldsymbol{y})^{T} \Sigma ^{-1} (\boldsymbol{x}-\boldsymbol{y})}$, where Σ is the variance-covariance matrix of ***x*** and ***y***. The variance-covariance matrix for uncorrelated variables (i.e. with zero covariances) is diagonal, and the Mahalanobis distance reduces to the normalized Euclidean distance. For uncorrelated variables with unit variances, the covariance matrix is the identity matrix and the Mahalanobis distance reduces to the ordinal Euclidean distance. Therefore, the Mahalanobis distance is equivalent to the Euclidean distance when the variables are standardized to Gaussian distributions with zero means and unit variances, through z-score transformations.

The $k-1$ nearest neighbours of every data point are first identified, thus defining *n* clusters of size *k*, where *n* is the length of each variable to be plotted. Working through each cluster one at a time, the algorithm then computes the coordinates of the centroid of its *k* associated data points. The coordinates of each centroid are estimated as the average of the coordinates of the *k* data points (i.e. the original data point and its $k-1$ nearest neighbours) in each dimension separately. For example in a 2-dimensional space, the *x*-coordinate of the centroid is the mean of the *x*-coordinates of the *k* data points and the *y*-coordinate of the centroid is the mean of the corresponding *y*-coordinates. The values of each of the original *n* data points are then replaced by the coordinates of their corresponding centroids. Finally, as the centroids tend to shrink towards the centre of mass of the data, we apply scaling to stretch them back to the observed variance of the original data, and re-centralize them back to the original mean (using the inverse of the z-score transformations). The scaling is performed by multiplying the centroids with a scaling factor, that is equal to the ratio between the standard deviation of the raw variable and the standard deviation of the calculated centroids.

Here, we use this procedure to produce the centroids which are considered as the anonymized values of the actual data and we display those centroids in different graphs. We use the value of $k=3$ in the algorithm of the *k*-nearest neighbours as a baseline value, but we vary this parameter in Sect. 4.4 to explore its effect on the data visualizations.

### Probabilistic anonymization

Probabilistic anonymization methods perturb data by the addition or multiplication of random stochastic noise [15, 25, 26]. A sufficient level of noise is selected to minimize the risk of re-identification of the original values from the perturbed data. However, an excessively high level of noise can so perturb the structure of the raw data that it can obscure important statistical properties [15]. In addition, because of the *law of large numbers*, the recurrent use of a probabilistic anonymization technique on a given dataset can reveal inference of the true records if the numeric seed that underpins the pseudo-random number generator that creates the embedded noise is different each time it is run. Therefore, to block potential inferential disclosure from a malicious attacker, the data custodian must fix the random number generator and must specify the level of noise in relation to their particular data situation. The seed for random number generation needs to be kept secret.

Here, we use the addition of random stochastic noise as a probabilistic way to generate disclosure protected data points to be used in graphical visualizations. Random noise is added to each variable separately before the generation of a specific graph. The noise added to a given variable follows a normal distribution with zero mean and standard deviation equal to a percentage *q* of the observed standard deviation of the variable. As a baseline level of noise we use the value of $q=0.25$, but we vary this parameter in Sect. 4.5 to explore its effect on the data visualizations. The value $q=0.25$ means that the standard deviation of the added noise is equal to 25% of the observed standard deviation of the original variable or equivalently its variance equals 6.25% of the original variance.

## Data

To illustrate the applicability of the three different approaches to anonymization in generating privacy-preserving graphs, we simulate three sets of two continuous variables each. We assume that each dataset represents 500 hypothetical individuals but we also analyse the performance of the techniques in smaller sample sizes (see Sect. 4.6). For the first dataset, *D*1, we simulate a normally distributed variable *X*, with mean equal to 10 and standard deviation equal to 0.5, ($X \sim N(10,0.5^{2})$), and a variable *Y*, which depends on *X* linearly with a relationship given by the formula $Y=X+\varepsilon _{1}$. The error term $\varepsilon _{1}$ follows the standard normal distribution $\varepsilon _{1} \sim N(0,1)$. For the second dataset, *D*2, we simulate a variable *X* which follows a log-normal distribution with mean equal to 0 and standard deviation equal to 0.5, ($X \sim \operatorname{Lognormal}(0, 0.5^{2})$), and a variable *Y*, associated with *X* by the non-linear relationship $Y=\log (X)+\varepsilon _{2}$. In this case, we assume that the error term follows a uniform distribution, $\varepsilon _{2} \sim U(0,1)$. For the third dataset, *D*3, we simulate a variable *X* which follows a beta distribution with shape parameters $\alpha =5$ and $\beta =2$, ($X \sim \operatorname{Beta}(5, 2)$), and an independent variable *Y*, which also follows a beta distribution with shape parameters $\alpha =\beta =0.5$, ($Y \sim \operatorname{Beta}(0.5, 0.5)$). The choice of those particular distributions and relationships is for demonstrating the performance of the techniques in their application to data exhibiting a wide range of statistical properties for the generated variables. The spectrum of statistical properties adopted for this article includes symmetric and skewed distributions, unimodal and bimodal distributions, unbounded, semi-bounded and bounded distributions, none, linear and non-linear relationships between pairs of variables, and relationships that invoke, or do not invoke assumptions of Gaussian errors and/or homoscedasticity. Nevertheless, we acknowledge that the performance of our methods could vary in other data settings we have not considered. For reproducibility purposes, we use R software (version 4.0.0) and we fix the seed of the pseudo-random number generator to the arbitrary value of 1234 (but ensure that it is set only once, at the top of a script). We consider the simulated variables to be the ‘real data’, we then generate the ‘anonymous data’ using the methods described above to produce graphical illustrations of the statistical and relational properties of both the real and anonymous data, in order to evaluate and compare the proposed techniques.

In this article we focus solely on the visualization of continuous data. Firstly, this is because continuous data are highly identifiable, as each individual has a unique numeric value for each attribute which is usually distinguishable from the values of the other individuals in a dataset. For categorical variables this is not the case, as the same value for more than one individual can belong to the same category. Secondly, because by definition categorical data are grouped into categories, there is often no requirement for an active procedure for disclosure protection: the only action required is to mitigate the risk of disclosure associated with categories with counts below an acceptable threshold. Thirdly, two of the anonymization techniques that we present here (deterministic and probabilistic anonymization), disturb the values of a given variable without consideration of underlying context. This can result in meaningless anonymized values when either method is applied to categorical data. For example, if we have a binary variable representing gender – let us assume that 0 denotes males and 1 denotes females – then either the deterministic or the probabilistic anonymization can replace values from any of the two categories to a numeric value within (or outside) the range of 0-1, which is meaningless when denoting the gender of an individual.

## Results

### Graphical displays for exploratory data analysis

Exploratory data analysis is used to summarize the basic features of the underlying data in a study. Graphical displays of exploratory statistics can describe univariate characteristics, such as the marginal distribution of a single variable, indicating its mean, skewness and kurtosis, or the dispersion of the variable indicating its range, quantiles, or central tendency (e.g. mean and median). The most common plots of univariate statistics are histograms and box plots. For bivariate (or multivariate) analysis, exploratory statistics are used to summarize the relationships between different variables which, can be displayed graphically in scatter plots, heat maps and contour plots. Here, we demonstrate how we can use the proposed anonymization methods to produce common graphical outputs useful in exploratory statistical analysis.

**Figure 1:**
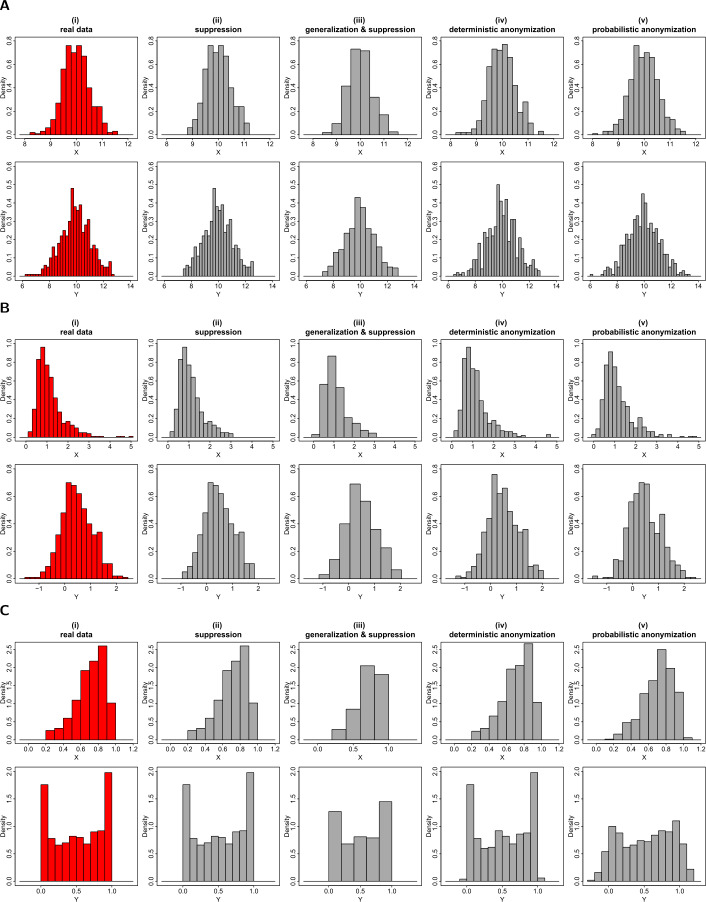
Privacy-preserving histograms. Figures (**A**), (**B**) and (**C**) show the histograms of *X* (top rows) and *Y* (bottom rows) from datasets *D*1, *D*2 and *D*3 respectively. From left to right we demonstrate (i) the histograms of the actual variables; (ii) the histograms of the variables after suppressing any bins with less than three counts; (iii) the histograms of the variables after generalizing the variables into bins based on wider intervals and suppressing any bins with less than three counts; (iv) the histograms of the scaled centroids of each 3-nearest neighbours; (v) the histograms of the variables with added noise of variance equal to 6.25% of the true variance. Note that the vertical axis represents the frequency density which is the frequency of each bin divided by its width

The first example is the graphical representation of the distribution of a variable in a histogram. To construct a histogram, the range of a variable is divided into a number of – typically – equal-sized intervals that define the widths of the histogram’s bins, and the frequency of the values falling in each of these intervals defines the height/area of the bin. If the range of a variable is divided into relatively small intervals, or if the variable includes some extreme outlier values, then a single or a small number of counts might fall into certain intervals. The histogram bars with low counts of observations are potentially disclosive, as one might be able to infer the small range within a certain individual-level record lies and, with potentially low uncertainty, to estimate its exact value. To ameliorate the risk of disclosure, the stated anonymization techniques are used to generate privacy-preserving histograms. Firstly, we apply the low counts suppression rule. Using this method, any histogram bar that includes less counts than an agreed threshold is suppressed from the plot. In panels (ii) of Fig. 1, we show the histograms of variables *X* and *Y* after suppressing any bars with less than three counts. Secondly, we apply generalization by increasing the width of the intervals that divide up the range of a variable, and redistributing the variable’s values into those new bins (see panels (iii) in Fig. 1). By increasing the width of the intervals, the number of the bins decreases, but also the probability of having bins with low counts of observations decreases. Any bins that still have sub-threshold counts will still be subject to the suppression rule. Thirdly, we apply deterministic anonymization, with the value of $k=3$ set for the number of nearest neighbours, and produce the histograms of the estimated scaled centroids (see panels (iv) in Fig. 1). Finally, we apply the probabilistic anonymization method by adding to each variable a normally distributed error with standard deviation equal to 0.25*σ* (where *σ* is the standard deviation of the original real variable) and generating histograms of the noisy data (see panels (v) in Fig. 1).

When the parameters of the anonymization techniques are selected carefully to mitigate the disclosure risk while preserving the shape of the underlying distributions, the generated histograms are both informative and secure. However, an unconsidered choice of the parameters can result in a distorted histogram. For example, let us assume that an analyst decides to use suppression with a big value of *k* (i.e. much greater than 3) to generate a privacy-preserving histogram of a variable with a long-tailed skewed distribution (like the log-normal distribution of variable *X* in Fig. 1(B)). Then, all the bins of the tail having lower counts than the selected threshold of *k* will be suppressed, and the displayed histogram will have a more symmetrical distribution obscuring the original asymmetrical shape. The shape can also be distorted when generalization creates unusually wide bins. A note here is that the bins do not have to be all of the same width. Given how we define our histograms, the area of the bar corresponds to the frequency, and therefore the widths of different bins can be manipulated in a way that will preserve the overall shape of the distribution, while hiding unique and outlying observations. Note that in Fig. 1 we display the density of each bin rather than the frequency, which is just the frequency divided by the width of the bin.

The other two anonymization techniques, deterministic and probabilistic, seem generally to perform well in generating privacy-preserving histograms. However, avoidance of selecting seriously inappropriate values for the control parameters in each specific data setting is particularly important for the probabilistic method. For example, large values can distort the apparent distribution. One example of such deformation, is the histogram of variable *Y* in panel (v) of Fig. 1(C), which fails to preserve the original bimodal nature of the variable (see histogram with red colour in panel (i) of Fig. 1(C)). In that example, a lower level of noise (i.e. noise with smaller variability) might be able to retain both the data utility and the individual privacy. Another disadvantage of these two methods is that they can both, in principle, generate anonymized values that fall outside the original (or theoretically defined) ranges characterising the underlying data. In most cases this is not a great problem as such values are generally very few in number. Furthermore, for the deterministic method it can only happen after final rescaling, when the underlying distribution generates an unusually large rescaling factor. Nevertheless, the fact that this can, in theory, occur should not be forgotten and it can potentially be problematic when the methods are applied to bounded (e.g. beta) or semi-bounded (e.g. log-normal) distributions. In such cases, some bins may be generated with values falling outside initial boundaries (see for example the bins with values below zero and above one in panels (iv) and (v) of Fig. 1(C)).

**Figure 2:**
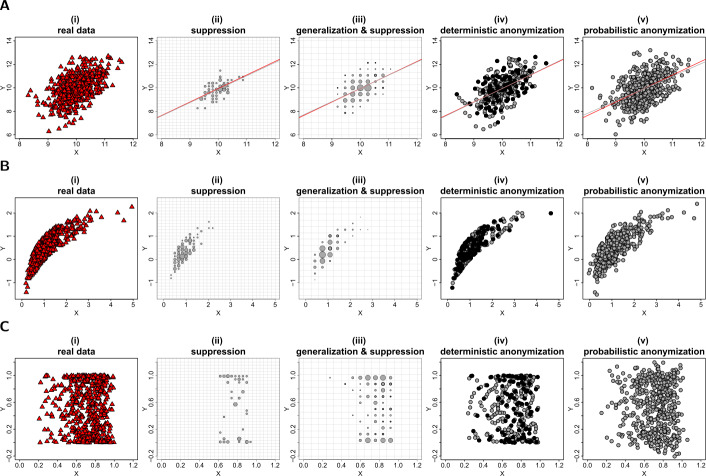
Privacy-preserving scatter plots. Figures (**A**), (**B**) and (**C**) show the scatter plots of *X* and *Y* from datasets *D*1, *D*2 and *D*3 respectively. From left to right we demonstrate: (i) the scatter plots of the actual variables; (ii) the scatter plots of the data aggregated in a 30 by 30 density grid matrix and suppressing any grids with density less than three counts; (iii) the scatter plots of the data aggregated in a 15 by 15 density grid matrix (i.e. additional generalization) and suppressing any grids with density less than three counts; (iv) the scatter plots of the scaled centroids of each 3-nearest neighbours obtained by deterministic anonymization; (v) the scatter plots of noisy *X* and *Y* obtained by addition of random stochastic noise in each variable, of variance equal to 6.25% of the true variability. Notes: Each data point in panels (ii)–(iii) is located at the center of the grid and its size corresponds to the number of observations in the grid. The grids are shown with transparent lines. Panels (ii)–(iii) in Figure (**A**) include the actual linear trend line of *X* and *Y* (red) and the weighted linear trend line of the *k*-anonymized data (grey). Panels (iv)–(v) in Figure (**A**) include the linear trend lines of actual (red) and anonymized (grey) *X* and *Y* variables. The black dots in panels (iv) indicate the positions where more than one centroids are identically placed

The second example of displaying exploratory statistics in graphs, is the visualization of data in scatter plots. A scatter plot is highly informative as it indicates the dependence (linear or non-linear relationship) between two variables and their correlation, if any. However, as a corollary, a scatter plot can also be potentially disclosive, as it provides a graphic representation of the exact coordinates of two variables from a set of data. A scatter plot can be rendered privacy-preserving, if the actual values of the two variables (particularly in outliers) can be concealed, while it remains informative if the graphic generated by the selected anonymization technique faithfully demonstrates the underlying statistical properties of the displayed variables. Figure 2 shows the privacy-preserving scatter plots of the data from the three datasets, generated using the *k*-anonymization (panels (ii) and (iii)), the deterministic anonymization (panels (iv)) and the probabilistic anonymization (panels (v)). The privacy-preserving scatter plots can be compared with the potentially-disclosive scatter plots of the raw data (panels (i)).

We mentioned earlier that the *k*-anonymization can be applied to plots where data are grouped either in bins (i.e. 1-dimension) or grids (i.e. 2-dimensions). So here, in order to produce the scatter plots of panels (ii) in Fig. 2, we first generate a 30 by 30 density grid matrix (or similarly a generalized 15 by 15 matrix for the scatter plots of panels (iii)) based on the ranges of the *X* and *Y* variables, and suppress any data that exist in grids with less than three counts. We then plot data points at the center of the remaining grids (i.e. those having more than three observations) and size the dots by the density of observations in their grid. It is therefore evident that the *k*-anonymization under-performs in the case of scatter plots, as there is a significant reduction of the information presented in the plots (i.e. more than half of the data are aggregated and/or suppressed).

On the other hand, the deterministic and, on some level, the probabilistic anonymization can retain the underlying dispersion of the original data in the 2-dimensional space. Based on a visual inspection, the statistical properties such as the mean, the variance, the covariance and the correlation of the variables appear to remain approximately stable, but the exact values of the raw data cannot be identified. A characteristic of the deterministic anonymization is that observations in a cluster of size *k*, which is isolated from the bulk of the data, share the same set of $k-1$ nearest neighbours and thus have identical centroids. It is therefore possible that in a scatter plot of scaled centroids, less points are visible when compared to the scatter plot of the corresponding raw data. The centroids that are located at the same positions with other centroids are shown by black dots in panels (iv) of Fig. 2. For the probabilistic anonymization, it is important to select an appropriate value for the parameter *q*, in order to keep the noisy data within an area that is not extended too much from the convex hull of the original values. This condition is important when the convex hull of the data defines the boundaries of bounded (or semi-bounded) distributions. A violation of that condition is observed in panel (v) of Fig. 2 where the noisy counterpart of variable *Y* has values out of its initial $[0-1]$ range.

Another graphical representation of data that is widely used is the heat map or image plot. A heat map represents a 2-dimensional display of values contained in a density grid matrix. Each grid is represented by different colours, indicating the density of the values in the grid. To generate a heat map plot of bivariate data, we first create their density grid matrix. We split the range of each variable into equal sub-intervals and enumerate the count of values falling in each grid on a 2-dimensional matrix (i.e. the density grid matrix). The pixels in the heat map plot then reflect the density in each cell of the grid, with a colour determined by the observed count. Using density grid matrices we can also display similar information in contour plots (i.e. topographic maps). A contour plot is a graphical display for representing a 3-dimensional surface by plotting constant *z* slices, called contours, on a 2-dimensional area. Given the values of *z*, which are the densities in the grid matrix, the lines that form the isometric contours connect $(x,y)$ coordinates where the same *z* values occur.

**Figure 3:**
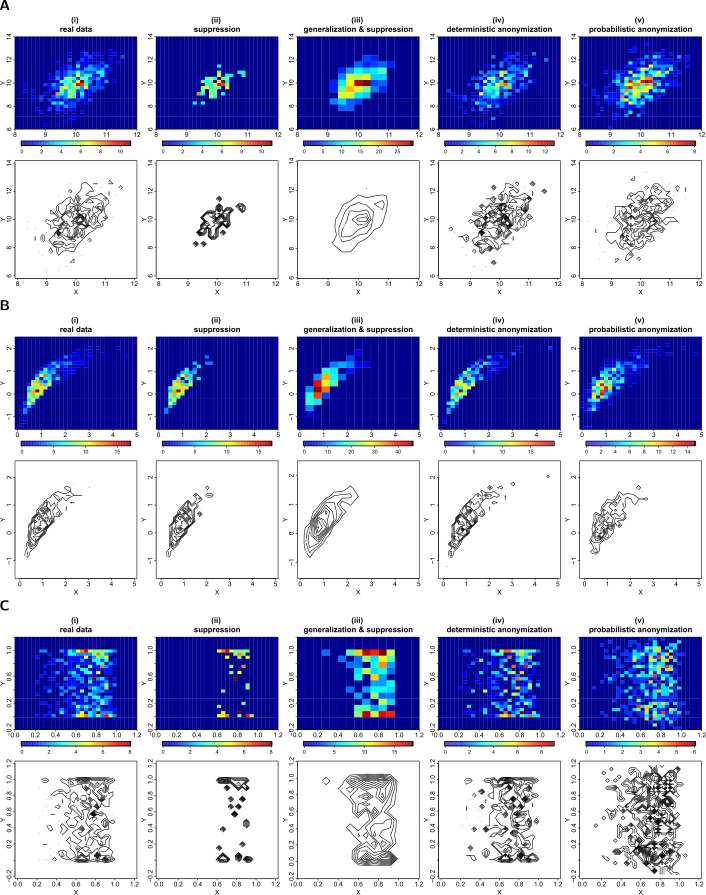
Privacy-preserving heat map and contour plots. Figures (**A**), (**B**) and (**C**) show the heat map (top rows) and contour (bottom rows) plots of *X* and *Y* from datasets *D*1, *D*2 and *D*3 respectively. From left to right we demonstrate the plots produced by: (i) the actual 30 by 30 density grid matrix; (ii) the actual 30 by 30 density grid matrix but suppressing any grids with density less than three counts; (iii) a generalized to 15 by 15 density grid matrix and suppressing any grids with density less than three counts; (iv) a 30 by 30 density grid matrix of deterministically anonymized variables using the value of $k=3$ when locating the *k*-nearest neighbours; (v) a 30 by 30 density grid matrix of probabilistically anonymized variables generated by adding random noise to the actual variables, of variance equal to 6.25% of their actual variance. Note that the colour scale differs between the different heat map plots

Panels (i) at the top rows of Figs. 3(A), 3(B) and 3(C) show the heat map plots of *X* and *Y* formed by using a 30 by 30 density grid matrix. In panels (ii), we show the privacy-preserving heat map plots produced using suppression of the grids with densities less than three counts (for example, in Fig. 3(A), panel (ii), 164 grids out of the 900 have been suppressed). In panels (iii) we display the privacy-preserving heat maps produced using a 15 by 15 density grid of the *X* and *Y* variables (i.e. generalization) and then suppressing any grids with densities less than three counts (for example, in Fig. 3(A), panel (iii), 42 out of 225 grids have been suppressed). Panels (iv) and (v) represent the privacy-preserving heat maps produced using the 30 by 30 density grid matrices of the deterministic and probabilistically anonymized data respectively. The bottom rows of Figs. 3(A), 3(B) and 3(C), show the contour plots produced from the same density grid matrices as the corresponding heat map plots.

**Figure 4:**
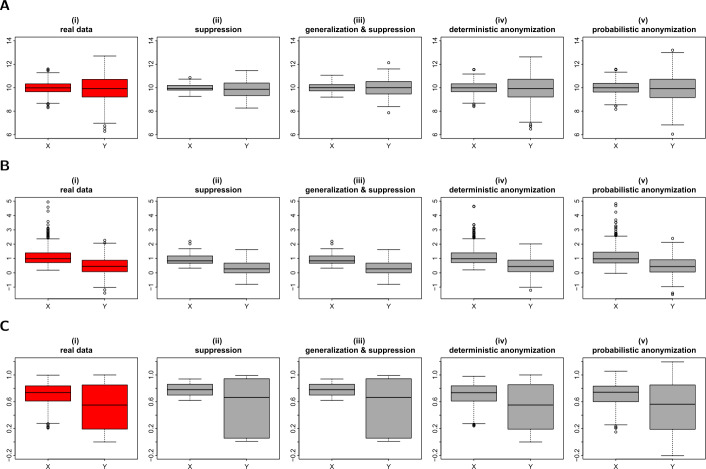
Privacy-preserving box plots. Figures (**A**), (**B**) and (**C**) show the box plots of *X* and *Y* from datasets *D*1, *D*2 and *D*3 respectively. From left to right we demonstrate: (i) the actual box plots of the variables; (ii) the data are aggregated in a 30 by 30 density grid matrix, any grids with density less than three counts are suppressed, and the box plots for the observations that are exist in the remaining grids are displayed; (iii) the data are aggregated in a 15 by 15 density grid matrix (i.e. further generalization), any grids with density less than three counts are suppressed, and the box plots for the observations that are exist in the remaining grids are displayed; (iv) the box plots of the scaled centroids of each 3-nearest neighbours obtained by deterministic anonymization; (v) the box plots of noisy variables obtained by addition of random stochastic noise in each variable, of variance equal to 6.25% of the true variability

The last example we present here, of a common visualization used on explanatory data analysis, is the box plot. A box plot visually shows the distribution of numerical data and skewness, by displaying the data quartiles (or percentiles) and averages. Potentially disclosive observations on a box plot are the outliers and the minimum and maximum points which define the ends of the whiskers. To protect such information from being disclosed, we apply the three anonymization techniques and generate privacy-preserving box plots. For the *k*-anonymization, we take the 30 by 30 density grid matrix of variables *X* and *Y* (or similarly the 15 by 15 matrix for the case of generalization), we suppress the grids with less than three counts, and then use the remaining values of each variable separately, to produce their privacy-protected box plots. For the deterministic and the probabilistic methods, we generate the scaled centroids and the noisy values respectively, and we then use those to produce the protected box plots. Figure 4 shows the box plots generated by suppression (panels (ii)), generalization and suppression (panels (iii)), deterministic anonymization (panels (iv)) and probabilistic anonymization (panels (v)) for the variables of the three simulated datasets.

From Fig. 4 we conclude that the *k*-anonymization suppresses the observations with high risk of identification (e.g. outliers), however it produces an undesirable information loss, as it tends to shrink the variables’ range (compare for example the whiskers of box plots of variable *Y* in panels (ii) and (iii) of Fig. 4(A) with the whiskers of the actual box plot shown in panel (i)). Another disadvantage of that method (as we also observed earlier in the case of histograms), is that it can suppress a significant number of observations and therefore can deform the underlying distribution and distort its statistical characteristics. One example of such distortion, is shown in the box plot of variable *Y* in dataset *D*3 for which its mean is shifted to the left under suppression (compare the mean of the box plots of *Y* between panels (i) and (ii) in Fig. 4(C)). This shift leads to the unwanted conversion of a symmetrical distribution to a less-symmetrical distribution.

On the other hand, the other two methods, the deterministic and the probabilistic anonymization, seem to perform better than the *k*-anonymization. One observation is that both of the methods keep displaying outlier points, however this is not a concern, as their location (and also their number) differs from the location of the outlier points in the original box plot. The only observed limitation of the probabilistic technique is that high level of noise might generate box plots with longer whiskers than the underlying whiskers, denoting an expanded range for the variable (see for example the box plot of variable *Y* in panel (v) of Fig. 4(C)).

### Regression plot diagnostics

In linear regression analysis, key statistical properties of the model, such as likelihood ratio tests, Wald tests, associated p-values, and the $R^{2}$ statistic, can provide information about the extent to which a model and its various components provide an adequate representation of its generating data. Amongst the approaches to exploring model fit, diagnostic plots are amongst the most important; they typically provide a quick and intuitive way to look at key aspects of model fit. For example, they can provide information about assumptions of linearity or constancy of variance. They can also reveal unexpected patterns in the data and can give insights on how the model structure may be improved. One important class of diagnostic plots makes use of regression residuals, representing the discrepancy between the observed outcome variable and the fitted model predictors in individual records. However, by combining the model covariates, the estimated regression coefficients, and knowledge of basic model structure (such as the link function), one can generate the predicted values and by then adding the vector of residuals to directly obtain the exact values of the outcome variable [27]. This is, by definition, disclosive. Furthermore, one of the most informative, and widely used, diagnostic tools is to plot residuals versus fitted (or predicted) values. With no other information at all, this can allow direct calculation of the actual values of the outcome variable. Because of the analytic value of such plots (whilst recognising the basic disclosure risk they create), we use the anonymization approaches to generate privacy- and information-preserving regression plot diagnostics. To do this, we first apply the regression model to the actual data and then use an anonymization process to anonymize the regression outcomes, and hence, allow visualization of the diagnostic plots, while mitigating the risk of disclosure. This approach can be used to visualize plots of residuals, metrics of leverage, influence measures and their relationships with fitted values.

**Figure 5:**
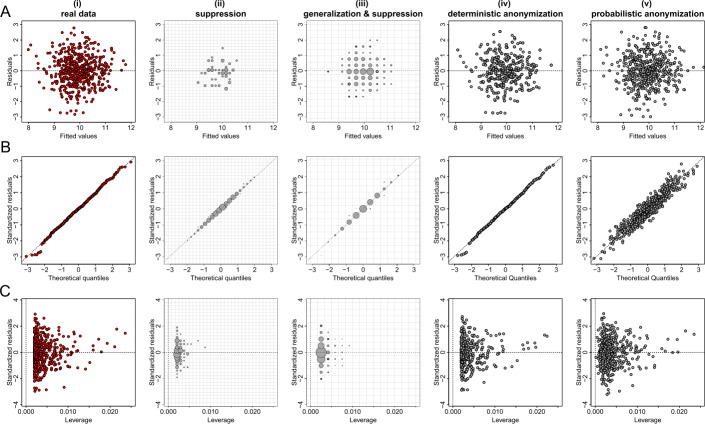
Privacy-preserving regression diagnostic plots of data from dataset *D*1. (**A**) Residuals against fitted values; (**B**) Normal QQ plots; (**C**) Residuals against leverage. From left to right we demonstrate the plots: (i) for real regression outcomes; (ii) the regression outcomes aggregated in a 30 by 30 density grid matrix and suppressing any grids with density less than three counts; (iii) the regression outcomes aggregated in a 15 by 15 density grid matrix and suppressing any grids with density less than three counts; (iv) the scaled centroids of each 3-nearest neighbours of the regression outcomes obtained by deterministic anonymization; (v) the noisy regression outcomes obtained by probabilistic anonymization. Note that for plots B and C we use the standardized residuals that are the residuals divided by their standard deviation

**Figure 6:**
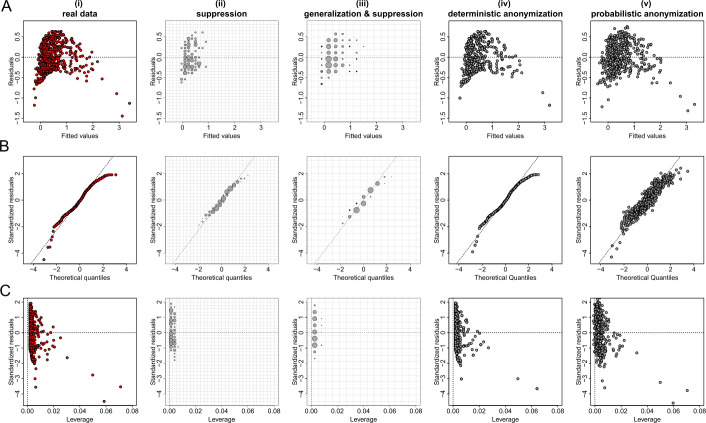
Privacy-preserving regression diagnostic plots of data from dataset *D*2. (**A**) Residuals against fitted values; (**B**) Normal QQ plots; (**C**) Residuals against leverage. From left to right we demonstrate the plots: (i) for real regression outcomes; (ii) the regression outcomes aggregated in a 30 by 30 density grid matrix and suppressing any grids with density less than three counts; (iii) the regression outcomes aggregated in a 15 by 15 density grid matrix and suppressing any grids with density less than three counts; (iv) the scaled centroids of each 3-nearest neighbours of the regression outcomes obtained by deterministic anonymization; (v) the noisy regression outcomes obtained by probabilistic anonymization. Note that for plots B and C we use the standardized residuals that are the residuals divided by their standard deviation

**Figure 7:**
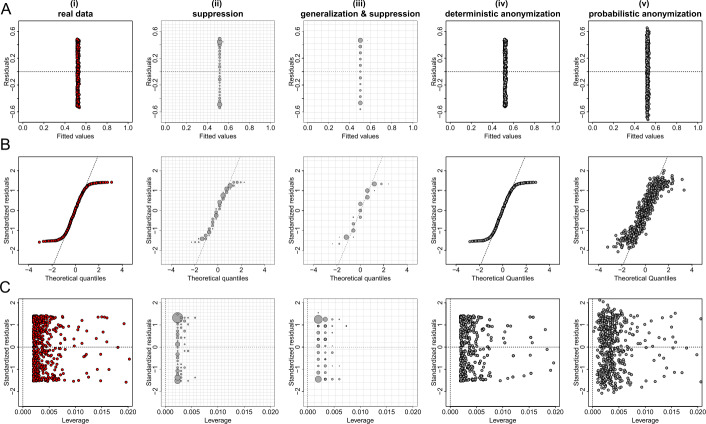
Privacy-preserving regression diagnostic plots of data from dataset *D*3. (**A**) Residuals against fitted values; (**B**) Normal QQ plots; (**C**) Residuals against leverage. From left to right we demonstrate the plots: (i) for real regression outcomes; (ii) the regression outcomes aggregated in a 30 by 30 density grid matrix and suppressing any grids with density less than three counts; (iii) the regression outcomes aggregated in a 15 by 15 density grid matrix and suppressing any grids with density less than three counts; (iv) the scaled centroids of each 3-nearest neighbours of the regression outcomes obtained by deterministic anonymization; (v) the noisy regression outcomes obtained by probabilistic anonymization. Note that for plots B and C we use the standardized residuals that are the residuals divided by their standard deviation

Figures 5(A), 6(A) and 7(A) show the plots of residuals against fitted values for the data from datasets *D*1, *D*2 and *D*3 respectively. For a number of classes of model, for example those with a Gaussian error and identity link (like the fitted model of the relationship between *X* and *Y* from dataset *D*1 which is given by $\hat{Y}=\beta _{0} + \beta _{1} X$), this type of plot can provide a convenient way to explore the basic assumptions of linearity and homoscedasticity (i.e. the residuals have constant variance) [28]. If the residuals are symmetrically distributed around the horizontal axis without nonlinear patterns and without a systematic trend in their dispersion, then it can reasonably be concluded that there is little or no evidence against the assumption that the regression relationship is linear and homoscedastic. Thus, Fig. 5(A) validates the assumptions of linearity and homoscedasticity, Fig. 6(A) provides evidence of a non-linear relationship between *X* and *Y*, and Fig. 7(A) indicates that there is no relationship between *X* and *Y* (i.e. the solution of a simple linear regression model is a horizontal line at $\hat{Y} \approx 0.5$).

Figures 5(B), 6(B) and 7(B) present normal quantile-quantile (QQ) plots for the residuals of the regression outcomes of data from datasets *D*1, *D*2 and *D*3 respectively. The QQ plots compare the observed distribution of residuals against a theoretical normal distribution by plotting their quantiles (percentiles). If the residuals do not seriously deviate from the line of identity, then we may conclude that they are consistent with a normal distribution. Thus, Fig. 5(B) confirms the normality of the residuals (as we have deliberately created the error term to follow a standard normal distribution – see Sect. 3), while Figs. 6(B) and 7(B) indicate that the residuals do not follow a normal distribution (for dataset *D*2 we already know that the residuals follow a uniform distribution – see Sect. 3). Finally, the plots in Figs. 5(C), 6(C) and 7(C) display the residuals against Cook’s distance which is a measure of local influence [29, 30]. Large Cook’s distance could be due to a large residual, high leverage or both. For the three examples shown in Figs. 5(C), 6(C) and 7(C), all cases are well inside the Cook’s distance lines which are not visible in the selected narrow window frames.

Those three regression diagnostic plots can be considered as scatter plots, as they present the dispersion of points in a 2-dimensional space. Therefore, the three anonymization techniques are applied here to generate privacy-preserving diagnostic plots for the regression model assumptions, in the same way as they used to produce privacy-protected scatter plots. Consequently, their performance is similar to what we observed in scatter plots. In other words, the *k*-anonymization causes a significant information loss (which is evident in almost all panels (ii) and (iii) of Figs. 5–7), while the deterministic and the probabilistic approaches preserve most of the actual characteristics of the plots. An exception is when the addition of random noise, through the probabilistic anonymization, on the points displayed in QQ plots, might obscure some characteristics of the actual trajectory of the points around the line of identity.

### The effect of parameter *k* of the *k*-anonymization process

**Figure 8:**
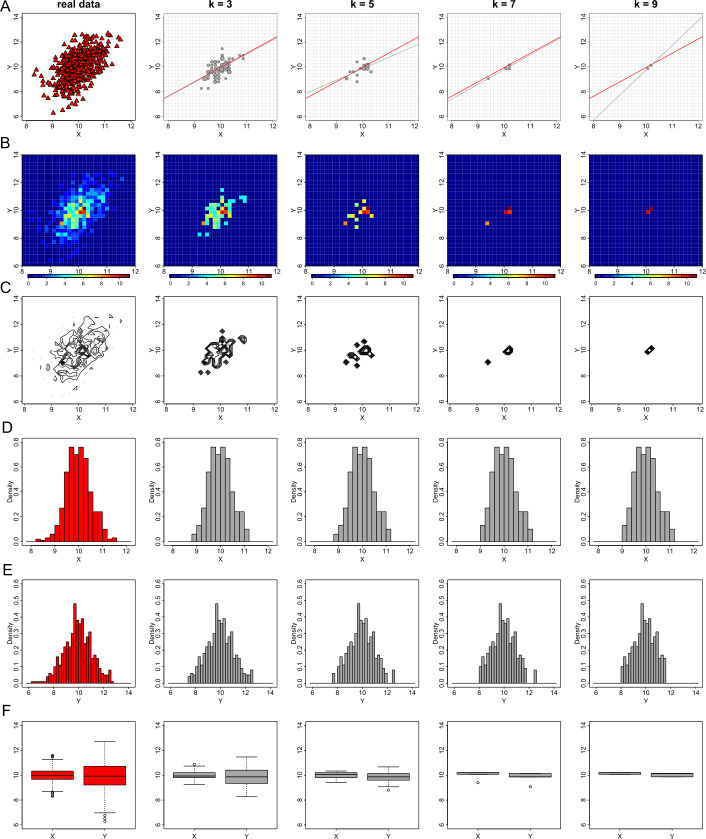
The effect of parameter *k* of the *k*-anonymization method on exploratory data visualizations. From left to right we demonstrate the effect of *k* for the values of 1 (this is equivalent to the real data), 3, 5, 7 and 9 on generating privacy-preserving scatter plots (**A**), heat map plots (**B**), contour plots (**C**), histograms of *X* (**D**) and *Y* (**E**), and box plots (**F**)

We assess the performance of the *k*-anonymization on generating privacy-preserving visualizations when varying the value of the parameter *k*. We use the values of *k* equal to 3, 5, 7 and 9 and produce visualizations of exploratory statistics and regression diagnostics. For simplicity, we only use the data from dataset *D*1. In Fig. 8 we show that when *k* increases the utility of the plots degrades with the exception of the histograms. As the two variables follow a normal distribution, the suppression rule suppresses – an equal number on average – bins from the two tails of their histogram. Thus, the histograms remain in some sense informative, even for $k=9$, as the symmetrical shape and the mean of the distributions remain stable. For all the other graphs (i.e. scatter, heat map, contour and box plots) there is an inevitable information loss, even when $k=3$, which increases with the increase of *k* and results in non-informative plots, which are useless for statistical analysis and derivation of conclusions.

**Figure 9:**
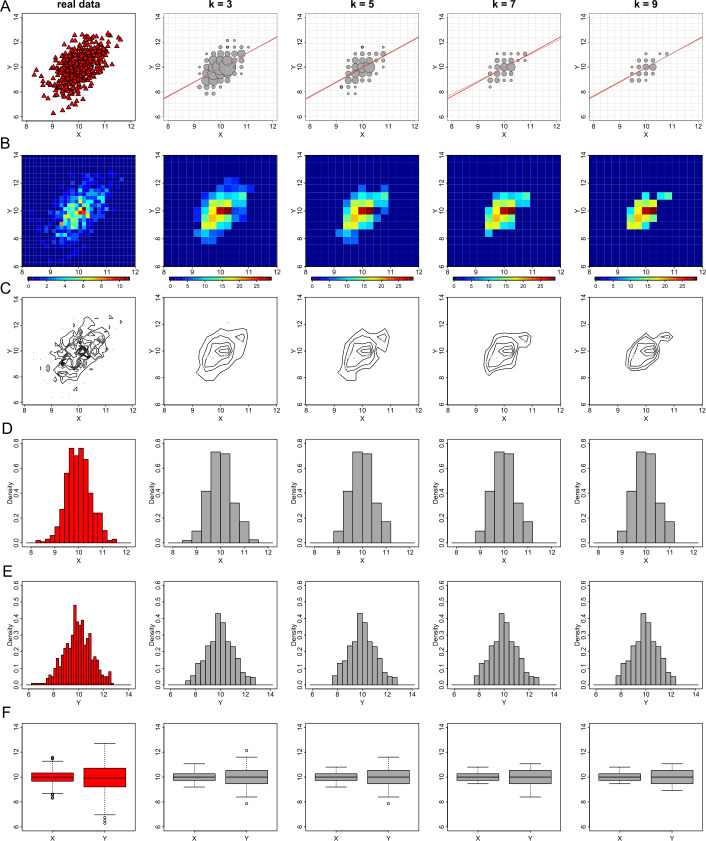
The effect of parameter *k* of the *k*-anonymization method on exploratory data visualizations with additional generalization. From left to right we demonstrate the effect of *k* for the values of 1 (this is equivalent to the real data), 3, 5, 7 and 9 on generating privacy-preserving scatter plots (**A**), heat map plots (**B**), contour plots (**C**), histograms of *X* (**D**) and *Y* (**E**), and box plots (**F**)

Moreover, when the *k*-anonymization is applied for the generation of privacy-preserving graphs, we need to examine not only the impact of the parameter *k* in the protection (and consequently the distortion) of the individual-level data, but also the level of generalization. For the scatter, heat map, contour and box plots of Fig. 8, we use a 30 by 30 density grid matrix and suppress any grids with less counts than the selected value of *k*. For the histograms, we divide the range of each variable in sub-intervals (which define the bins) of width equal to 0.2, and then suppress any bins with less than *k* observations. In Fig. 9, we present the same plots as those in Fig. 8, with the same variation of the value of parameter *k*, but with a doubled level of generalization. In other words, we use a 15 by 15 density grid matrix for the generation of scatter, heat map, contour and box plots and bins of width equal to 0.4 for the generation of histograms. As a result, there is again a loss of information on the graphical outputs, however this loss is lower than the loss we observed previously.

**Figure 10:**
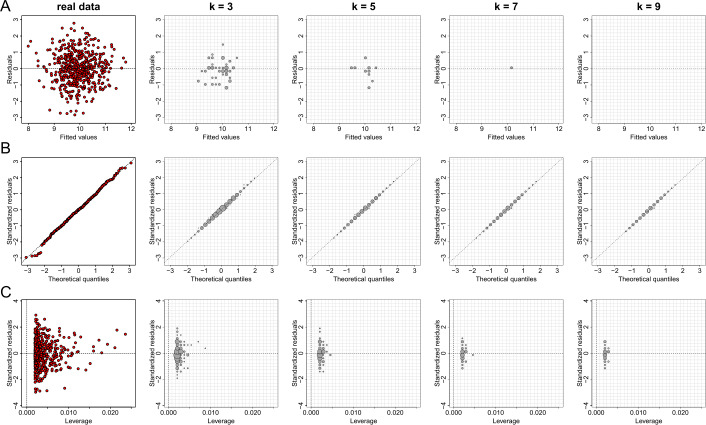
The effect of parameter *k* of the *k*-anonymization method on regression plot diagnostics. From left to right we demonstrate the effect of *k* for the values of 1 (this is equivalent to the real data), 3, 5, 7 and 9 on generating privacy-preserving residuals vs fitted values plots (**A**), normal QQ plots (**B**), and standardized residuals vs leverage (**C**)

**Figure 11:**
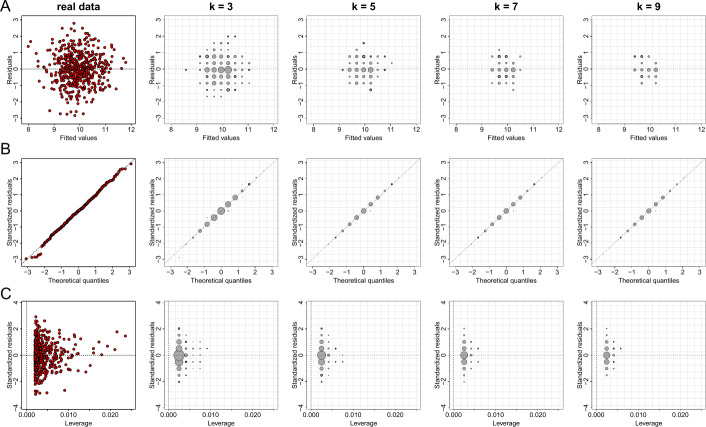
The effect of parameter *k* of the *k*-anonymization method on regression plot diagnostics with additional generalization. From left to right we demonstrate the effect of *k* for the values of 1 (this is equivalent to the real data), 3, 5, 7 and 9 on generating privacy-preserving residuals vs fitted values plots (**A**), normal QQ plots (**B**), and standardized residuals vs leverage (**C**)

Using the same values of parameter *k* and the same considerations of generalization, we reproduce the regression diagnostic plots for the regression outcomes of the linear model between the variables *X* and *Y* of dataset *D*1. As those graphs are of the same type as scatter plots, the information loss is significant and thus the statistical utility of the plots is negligible (see Figs. 10 and 11).

### The effect of parameter *k* of the deterministic anonymization process

**Figure 12:**
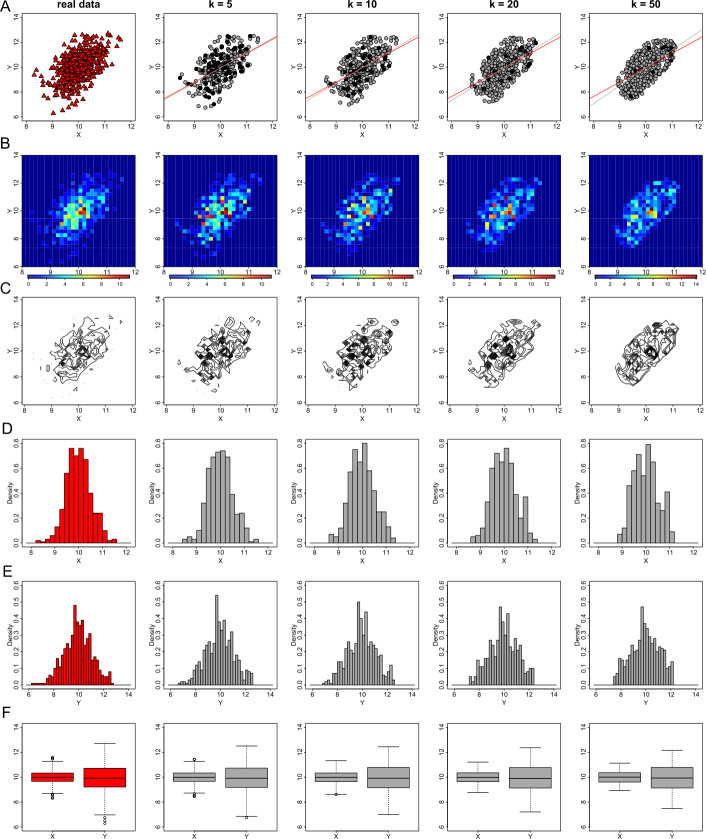
The effect of parameter *k* of the deterministic anonymization method on exploratory data visualizations. From left to right we demonstrate the effect of *k* for the values of 1 (this is equivalent to the real data), 5, 10, 20 and 50 on generating privacy-preserving scatter plots (**A**), heat map plots (**B**), contour plots (**C**), histograms of *X* (**D**) and *Y* (**E**), and box plots (**F**)

We analyse the performance of the deterministic anonymization on generation of privacy-preserving visualizations, with different values of the parameter *k*. Here we use the values of *k* equal to 5, 10, 20 and 50. Figure 12 displays the generated protected graphs that can be used for explanatory statistical analysis. We observe that, as the value of parameter *k* increases, the anonymized data are shifted to the center of their mass (compare panels from left to right in Fig. 12(A), (B), and (C)). This behaviour causes some level of information loss, as the trend line that best describes the linear relationship between the variables *X* and *Y*, slightly deviates from its actual position (compare red and grey trend lines in Fig. 12(A)). However, this information loss is significantly lower than the information loss which can be introduced through the use of the *k*-anonymization. Another observation is that the number of centroids that are placed in identical positions (black dots in Fig. 12(A)) is decreasing as the value of *k* increases.

**Figure 13:**
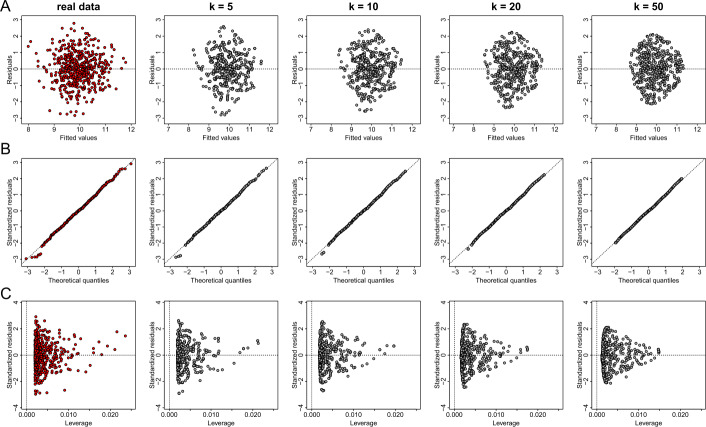
The effect of parameter *k* of the deterministic anonymization method on regression plot diagnostics. From left to right we demonstrate the effect of *k* for the values of 1 (this is equivalent to the real data), 5, 10, 20 and 50 on generating privacy-preserving residuals vs fitted values plots (**A**), normal QQ plots (**B**), and standardized residuals vs leverage (**C**)

The observation that the centroids tend to be “attracted” towards their center of mass as the value of *k* increases, is also observed in the residuals versus fitted values, and the residuals versus leverage plots (Figs. 13(A) and (C)). This attraction does not change considerably the structure of the displayed points on the graphs (at least for the case of the bivariate normal distribution), and therefore valid conclusions about the linearity of the model, the homoscedasticity of the errors and the existence of any influential points could be derived. However, for big values of *k* (e.g. greater than 10), the extreme quantile values of the residuals presented in QQ plots disappear (see for example the right panel in Fig. 13(B)), and therefore such plots are not informative for the diagnosis of the residuals’ normality. However, it should be noted that small values of *k* (e.g. values between 3 to 10) can be used to generate privacy- and utility-preserving visualizations.

### The effect of parameter *q* of the probabilistic anonymization process

**Figure 14:**
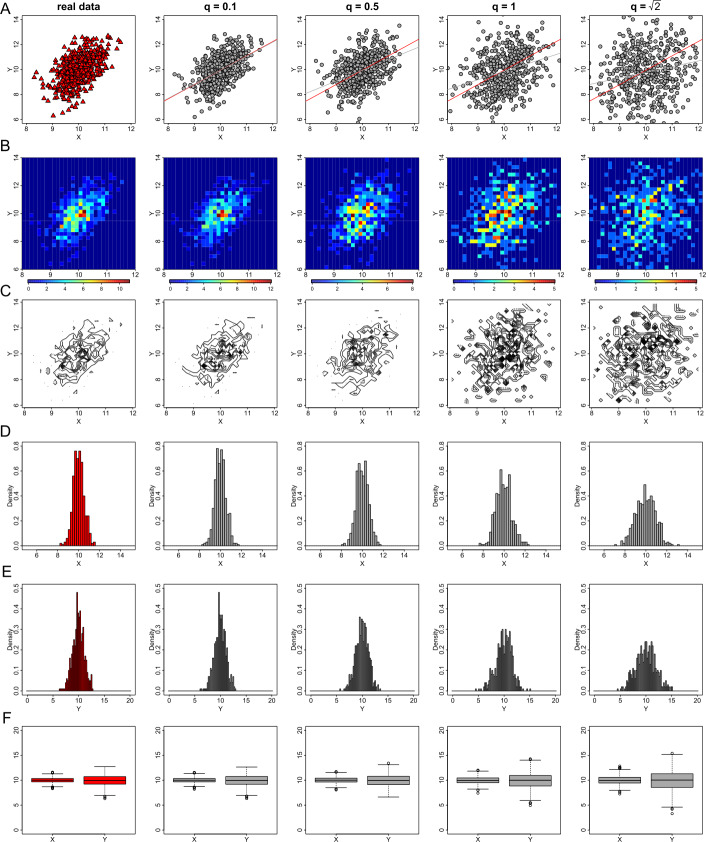
The effect of parameter *q* of the probabilistic anonymization method on exploratory data visualizations. From left to right we demonstrate the effect of *q* for the values of 0 (this is equivalent to the real data), 0.1, 0.5, 1 and $\sqrt{2}$ on generating privacy-preserving scatter plots (**A**), heat map plots (**B**), contour plots (**C**), histograms of *X* (**D**) and *Y* (**E**), and box plots (**F**)

A sensitivity analysis is also performed for different levels of noise added to the underlying variables through the probabilistic anonymization. We select the values of parameter *q* equal to 0.1, 0.5, 1, and $\sqrt{2}$. When $q=1$ the variance of the added noise is equal to the variance of the real variable; when $q=\sqrt{2}$, the variance of the noise is double the variance of the real variable. Figure 14 shows the privacy-preserving data visualizations for exploratory analysis, generated by the probabilistic anonymization with variation of parameter *q*. In this Figure, we observe an outspread of the data points when *q* increases. This was an expectation due to the increase in the variability of the data. Therefore, large values of *q* (e.g. greater than 1) generate large noise, which distort the displayed data and misrepresent the underlying statistical properties. For example, the heat map plot on the right panel of Fig. 14(B), obscures the apparent correlation of the variables which is observed in the heat map plot of the actual variable on the left panel. Similar deformation is observed in the generated histograms (Figs. 14(D) and (E)), which still indicate the symmetry of the normal distributions and retain their average, but expand their variance.

**Figure 15:**
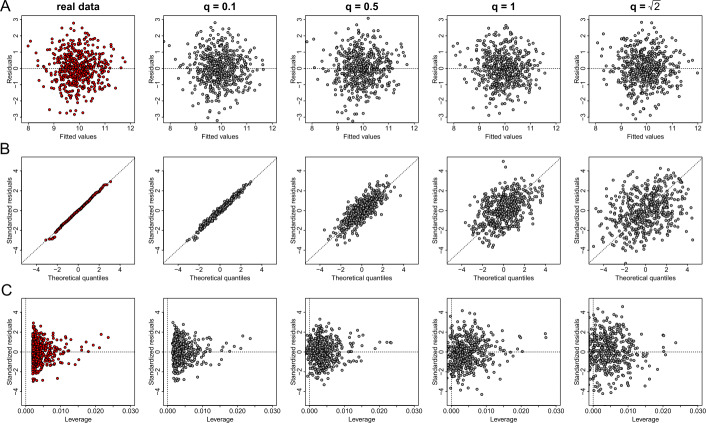
The effect of parameter *q* of the probabilistic anonymization method on regression plot diagnostics. From left to right we demonstrate the effect of *q* for the values of 0 (this is equivalent to the real data), 0.1, 0.5, 1 and $\sqrt{2}$ on generating privacy-preserving residuals vs fitted values plots (**A**), normal QQ plots (**B**), and standardized residuals vs leverage (**C**)

In Fig. 15 we show how the variation of parameter *q* affects the utility of regression diagnostic plots. Here, as we use the two normally distributed variables from dataset *D*1, the generated plots of the residuals versus fitted values (Fig. 15(A)), preserve the correlation between the two quantities (even for $q=\sqrt{2}$), as the independent noise added to each vector follows a normal distribution. Therefore, valid inferences for the model linearity and homoscedasticity can be derived. On the other hand, the addition of random noise on the quantiles of residuals (Fig. 15(B)), and on the leverage and residuals (Fig. 15(C)) deform the underlying characteristics of the corresponding plots when *q* increases. Thus, the privacy-preserving QQ plots can not provide evidence for the errors’ normality. Similarly, the noisy points displayed in the residuals versus leverage plots spread on the vertical direction of the graph as *q* increases, hence may produce leverage points which do not exist in the real data.

### The performance of the techniques in different sample sizes

**Figure 16:**
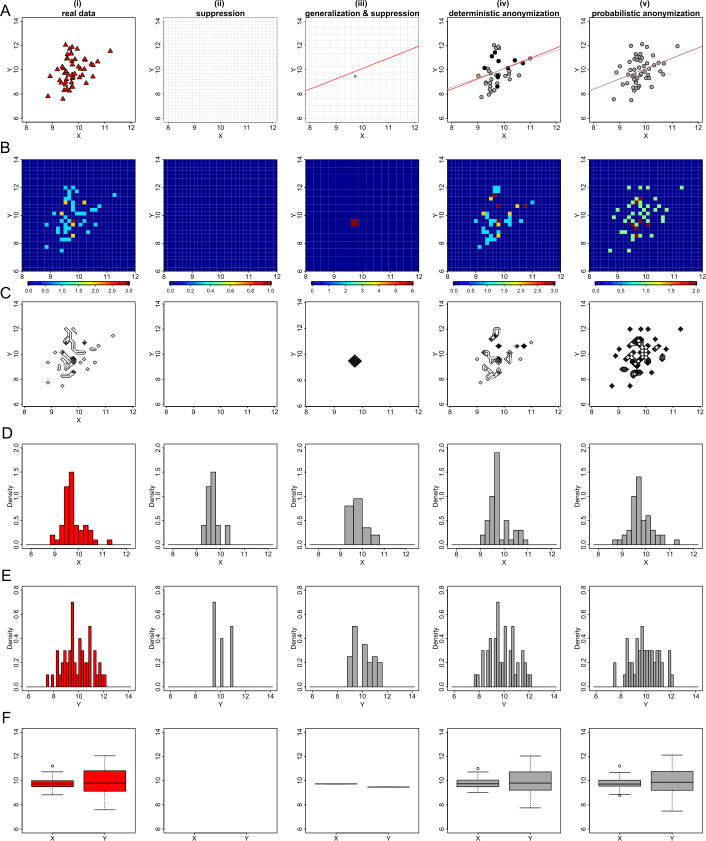
The performance of the techniques applied in a sample of 50 observations. From top to bottom we demonstrate the scatter plots (**A**), heat map plots (**B**), contour plots (**C**), histograms of *X* (**D**) and *Y* (**E**), and box plots (**F**) for real data (i), suppressed data (ii), generalized and suppressed data (iii), deterministically anonymized data (iv) and probabilistic anonymized data (v)

**Figure 17:**
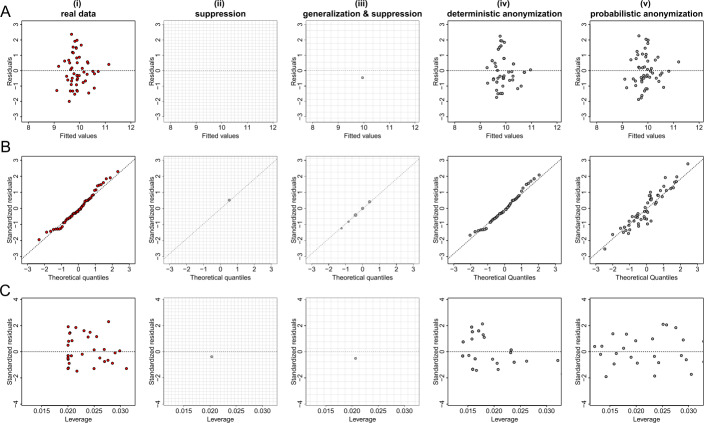
The performance of the techniques applied in a sample of 50 observations. From top to bottom we demonstrate the residuals vs fitted values plots (**A**), normal QQ plots (**B**), and standardized residuals vs leverage plots (**C**) for real regression outcomes (i), suppressed regression outcomes (ii), generalized and suppressed regression outcomes (iii), deterministically anonymized regression outcomes (iv) and probabilistic anonymized regression outcomes (v)

**Figure 18:**
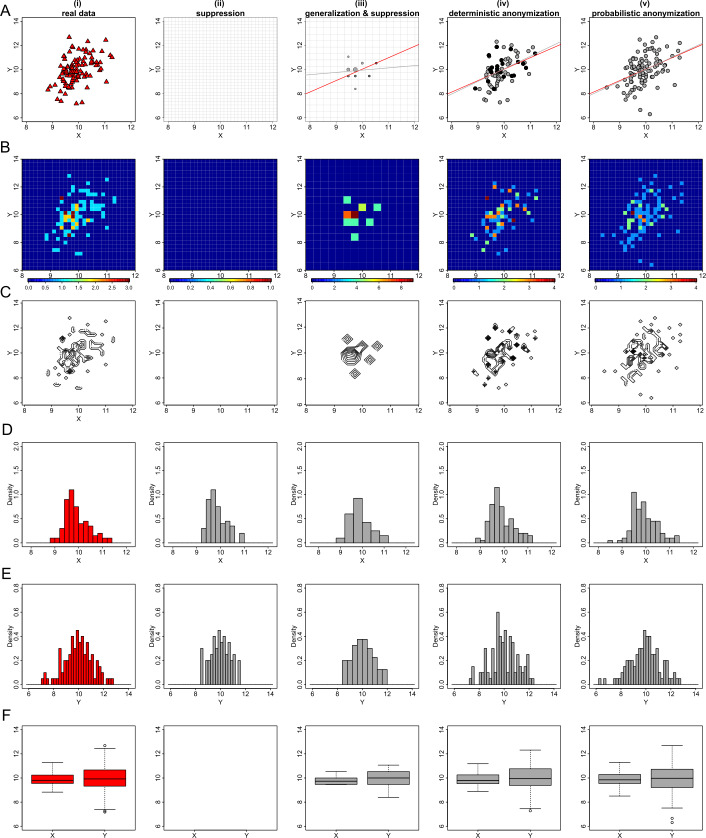
The performance of the techniques applied in a sample of 100 observations. From top to bottom we demonstrate the scatter plots (**A**), heat map plots (**B**), contour plots (**C**), histograms of *X* (**D**) and *Y* (**E**), and box plots (**F**) for real data (i), suppressed data (ii), generalized and suppressed data (iii), deterministically anonymized data (iv) and probabilistic anonymized data (v)

**Figure 19:**
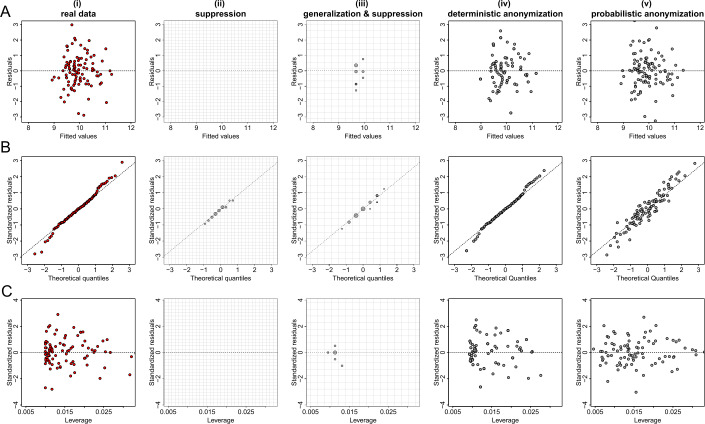
The performance of the techniques applied in a sample of 100 observations. From top to bottom we demonstrate the residuals vs fitted values plots (**A**), normal QQ plots (**B**), and standardized residuals vs leverage plots (**C**) for real regression outcomes (i), suppressed regression outcomes (ii), generalized and suppressed regression outcomes (iii), deterministically anonymized regression outcomes (iv) and probabilistic anonymized regression outcomes (v)

**Figure 20:**
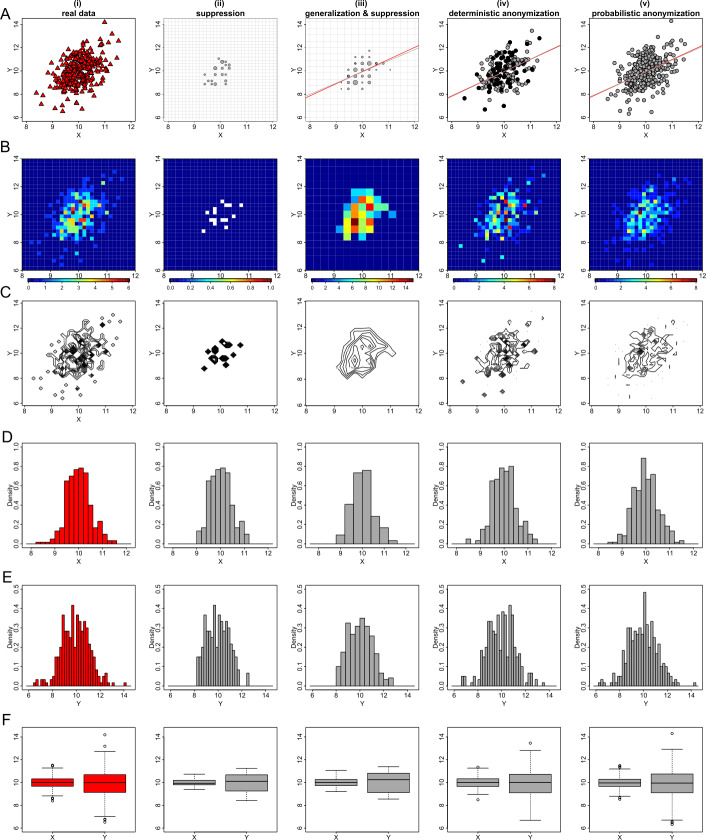
The performance of the techniques applied in a sample of 300 observations. From top to bottom we demonstrate the scatter plots (**A**), heat map plots (**B**), contour plots (**C**), histograms of *X* (**D**) and *Y* (**E**), and box plots (**F**) for real data (i), suppressed data (ii), generalized and suppressed data (iii), deterministically anonymized data (iv) and probabilistic anonymized data (v)

**Figure 21:**
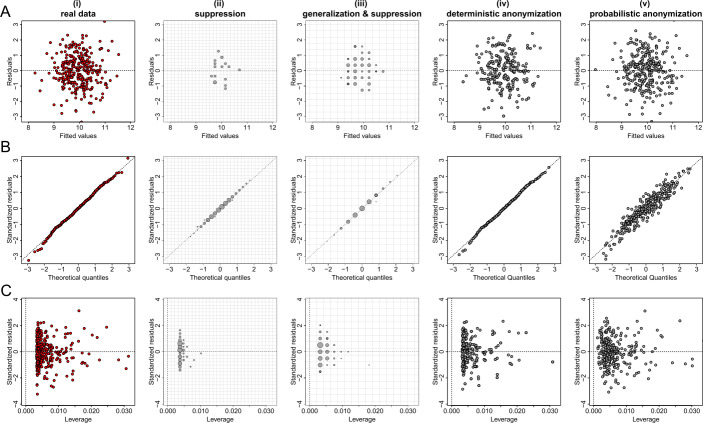
The performance of the techniques applied in a sample of 300 observations. From top to bottom we demonstrate the residuals vs fitted values plots (**A**), normal QQ plots (**B**), and standardized residuals vs leverage plots (**C**) for real regression outcomes (i), suppressed regression outcomes (ii), generalized and suppressed regression outcomes (iii), deterministically anonymized regression outcomes (iv) and probabilistic anonymized regression outcomes (v)

We acknowledge that it is a difficult task to protect the privacy of individuals in datasets of small sample sizes, without losing information. Here, we use the three anonymization techniques to generate privacy-preserving plots for data from small sample sizes to illustrate their performance. We simulate three sets of two normally distributed variables, each having the same statistical characteristics as those of dataset *D*1, with 50, 100 and 300 observations respectively. Figures 16, 18 and 20 show the generated privacy-preserving plots of exploratory statistics, and Figs. 17, 19 and 21 show the generated privacy-preserving regression diagnostic plots for each sample size respectively. As we can see from those six Figures, the *k*-anonymization can not be used on data from small sample sizes, because it eliminates too much information from the plots. The deterministic anonymization performs quite well in almost all of the instances, while the probabilistic anonymization also performs well, but a careful selection of the parameter *q* is crucial to prevent unwanted over-anonymization.

## Discussion

In this article we demonstrate how data anonymization techniques can be used to protect the privacy of data displayed in graphical outputs. The efficacy of such techniques may be assessed by their ability to simultaneously restrict: (a) the disclosure risk – i.e. the risk that a user may be able, accidentally or maliciously, to identify sensitive individual-level information from a graph, which may reveal either identity or important characterising information; and (b) the information loss arising from the process of anonymization. The disclosure limitation methods described in this paper can help address both of these issues, thereby facilitating the undoubted scientific benefits of presenting data in visual formats that are easily understood while protecting against the risks of disclosing identity or sensitive information. However, we note that even when the generation of a graph obeys a seemingly secure procedure, information that is made publicly available through other statistical research (including other data visualizations and statistical outcomes) or by the individual him/herself, may provide enough clues to permit a malicious and determined attacker to infer sensitive personal information. The primary objective of our work may therefore be viewed as making any such inference difficult, and wherever a formal log is kept of all analytic procedures undertaken (e.g. as in DataSHIELD [31, 32]) to make it easier to detect that a deliberate attempt has been made to infer identity or associated information, thereby opening up the option to sanction the action and/or to limit the perpetrator’s future access to sensitive data. However, because of the capacity to link data from multiple sources, it would be desirable for the possible need for active privacy-preservation to be considered whenever sensitive data objects – including visualizations – are made available (e.g. [33]) and objects such as these ought ideally to be protected not only by data-privacy policies and procedures but also under a broader meta-privacy framework [34].

Deciding and agreeing upon a framework for mitigating disclosure risk associated with visually represented data should be seen as a shared responsibility of data custodians and the analysts who will create the visualizations. They should make decisions based on a rational assessment of the real risks that are typically highly context dependent. The various methods we present have different strengths and weaknesses. The final choice of method should depend on a rigorous assessment of the real risks of disclosure – and its seriousness should it occur – versus the magnitude of the disbenefits of applying privacy protection which not only include increased time and effort but also the potential information loss that may be consequent upon it. All of these issues must be weighed up and applied to the specific data/analysis context that applies. In many cases data protection through a judicious application of suppression and generalization may be enough to mitigate the risk of disclosure and preserve data utility, while in other contexts deterministic or probabilistic anonymization may be preferable (particularly on scatter plots).

### *k*-anonymization

The *k*-anonymization method significantly reduces the disclosure risk from graphical displays, but it generally degrades the information content and, not infrequently, this may significantly impair data utility. For instance consider the apparent loss of information in the heat maps shown in Fig. 3 where much of the peripheral data are lost. The utility loss from this technique is clearly associated with the selection of the value of *k*. There is no consensus on the optimal value for the parameter *k*. Commonly used values are 3, 5 or 10 but there is much debate over these values (see also a brief analysis on the impact of the variation of that parameter in Sect. 4.3). Furthermore, even if it could be proven that one choice of *k* was, in some sense, optimal in one context, that would not imply that it was the best in other contexts. A higher value of *k* corresponds to a stricter privacy standard, because more individuals are then required to exhibit any given combination of identity-revealing traits. Any value less than three should probably be avoided because with just two observations in a subgroup, precise values can be inferred directly from a combination of mean and standard deviation (or variance) in that subgroup, and these are often reported in data summaries. In some cases however, there is little choice but to adopt the value of $k=1$ (i.e. no suppression or generalization is to be applied, particularly where rare combinations of attributes have an important role in the research questions and analysis. This implies that *k*-anonymization is not the method of choice for studies of rare diseases, conditions or health outcomes. Under such circumstances approaches based on deterministic or probabilistic anonymization are likely to be preferable as they can conceal unique combinations and outlier points without actually suppressing them. This logic implies that it would be very unwise to bow any top-down pressure to adopt *k*-anonymization as the norm, particularly if this also involved specification of a specific value of *k* to be used in all circumstances. This could lead to serious scientific costs and yet the decision could not, in principle, be based on a rational scientific logic. Additional arbitrary rules have to be applied when using *k*-anonymization because there is no optimal – or even best-practice – procedure that can be followed to take a given dataset and to convert it into a *k*-anonymous form. This implies that implementation will typically differ from study to study and this may lead to discrepant results.

In practice, *k*-anonymization typically *over-anonymizes* data [35] an impact characterised by substantive distortion of the data, including substantial information loss, and reduction of the utility of the data for subsequent analysis and visualizations [36]. This problem is most pronounced when *k*-anonymization is applied to a dataset with a small sample size (see Sect. 4.6) or to high-dimensional data [37]. The suppression of a large number of records threatens the integrity of a dataset and can produce systematic biases when values are eliminated disproportionately to their original distribution. At the same time, aggregated values formed through generalization are typically less informative than granular values, and this also impairs the quality of inference based on resulting analyses. For example, aggregated values preclude the fit of a linear regression based on generalized numerical attributes [38].

### Probabilistic anonymization

Under probabilistic anonymization the efficacy of the disclosure control, the magnitude of the corresponding loss of information, and the balance between them, depend not only on the variance of the added noise, but also – in any specific case – on the particular seed used to start the pseudo-random number generator. An important implication is that, the seed must be kept confidential, as users could otherwise generate the same set of errors and by subtracting them from the ‘noisy’ data reverse the original anonymization. Perhaps less obviously, it is also important not to release data anonymized multiple times using different seeds, as the errors terms that protect against disclosure, will ultimately tend to zero if one takes the mean of numerous realizations of the noise-augmented data.

Under some circumstances, like *k*-anonymization, probabilistic anonymization can disturb the statistical properties of the underlying data and inferences based on their graphical visualization. For example, for a QQ plot where the original data points are correctly distributed along a coherent line, the addition of random noise to the data both in the horizontal and vertical dimensions (as the method dictates) will convert the perfect line (in which *x* and *y* values have the same ordering) into a random stochastic scatter plot, which could result in misleading conclusions. For example, an analyst seeing the noise-augmented data displayed in the right panels of Fig. 15(B), might conclude that the assumption that the residuals are normally distributed is violated, however this is clearly not true if we look at the corresponding Figure of the real data (left panel of Fig. 15(B)). This happens because probabilistic anonymization, as described, adds vertical and horizontal noise to each data point on the plot *after it has been constructed* and each individual point may therefore move in any direction within a circle of radius proportional to the variance of the added noise. We adopt this particular approach to noise augmentation because it can be applied to *any* scatter plot. But in the particular case of a QQ plot this changes the nature of the plot from what is usually expected – a coherent line with *x* and *y* values in the same order – into a stochastic scatter plot. If the variance of the noise is large this might easily confuse an analyst with limited experience of probabilistic anonymization. In the particular case of the QQ plot it would be possible to add the probabilistic noise in a different manner: specifically, to add one dimensional noise to the variable being explored before, rather than after, creating the QQ plot. This would indeed produce a coherent line that would therefore be familiar to any analyst. But if the added noise has a very large variance, it is the noise – not the original data – that will dominate the distribution of noise-augmented data and because the noise is normally distributed, the greater the noise that is added, the closer the resultant variable will appear to being normally distributed. Because the approach we describe effectively applies the anonymization process after the plot has been constructed, it can be applied to *any* scatter plot and this is why we advocate it. However, an alternative anonymization protocol may sometimes be viewed as preferable but, if so, the QQ plot example demonstrates that one must properly understand the impact of the noise augmentation to ensure that it does not do something unexpected. In some circumstances, higher level of noise may deliberately be added to outliers or to points with high leverage in contrast to data points lying in the body of the data [39]. This can strengthen the control of disclosure risk for those particular points that are most likely to be identified. However, because these same points may be reflective of those very relationships in the data that are meant to be teased out by the graphical representation, care must be taken when doing this and when interpreting privacy-preserving graphics generated in this manner.

As an approach to statistical disclosure control, probabilistic anonymization offers an important benefit. That is, an analyst who has permission to work with the data can be provided with sufficient information (e.g. the variance of the noise) to correctly adjust inferences based on the noisy data to recover the underlying data structure and thereby to produce consistent parameter estimates [15]. This is not the same as removing every error term representing the noise – that would be fully disclosive – but rather through the use of statistical techniques for fitting models with measurement errors [40].

### Deterministic anonymization

In terms of minimizing the loss of utility while offering robust privacy-preservation, deterministic anonymization appears to be a particularly promising approach to the generation of disclosure-controlled data visualizations that faithfully retain important characteristics of underlying statistical properties. Although it could in principle perform less well on other classes of dataset, amongst the broad spectrum of examples we tested, there was no example where a primary feature of the original data was lost, or an artifact was created.

As for *k*-anonymization and probabilistic anomyization, the chosen value of *k* again impacts on both the quality of disclosure control and the potential for information loss or data distortion. The only recommendations we make are that *k* should be three or more and less than or equal to the number of the data points minus three. This range is chosen to make it difficult to confidently infer the value of any individual data points from deterministically anonymized data. In fact, given *k* is three or more, we have found no example to date where it would be possible to infer individual data points without knowing more about the underlying distribution of the data, e.g. the original relative distances between observations in a particular cluster of outliers. Nevertheless, we are aware that such settings might exist and when data are particularly sensitive it may therefore be preferable to increase *k*. As the value of *k* increases the disclosure risk decreases while details in the observed distribution gradually fade. However, major features of the individual variables and relationships between variables are well preserved – hence our reason for recommending this approach. That said, as *k* increases or when data are structured in discrete clusters that happen to be of length *k*, the centroids generated for each cluster overlap and the number of observations that are apparently visible in the graphs is less than the length of original data.

One fortuitous characteristic of deterministic anonymization is that it further shifts outlying points because of the final scaling procedure and this adds to the difficulty of any attempt to infer the position of the original data points. On the other hand, we have found no setting in which this additional unpredictability has substantially degraded the true information content in a deterministically anonymized visualization. As we note earlier, if the scaling factor is large enough, it is in fact possible for some outlying and influential points to be shifted out of the convex hull of the original data and users need to be aware that this is not an error – just a feature of the approach – and we have again found no setting in which this makes a visualization misleading.

### Conclusion

The work described in this paper contributes a variety of techniques that can be used to render informative visualizations whilst protecting the confidentiality of data. These methods can be used across a range of disciplinary domains wherever data are viewed as sensitive either because of concerns relating to information governance and/or data ethics or because of their value in terms of intellectual property. These approaches are therefore of interest to a wide range of data stakeholders: not just data analysts, but also data custodians, study participants and the general public who all have an interest in ensuring that data are used as widely as possible in ways that are of value to society and yet protect their confidentiality. The development of privacy-preserving data visualizations has important applications in scientific research including visual data exploration, visual representations of descriptive and summary statistics, and regression plot diagnostics. In order to encourage and facilitate the widespread adoption of techniques such as these, there is a need to develop new data science methodologies and infrastructures that incorporate them. Such solutions have the potential to enhance the discoverability and utility of individual-level data across all disciplines where data can be sensitive. These techniques can help to bolster public support for the collection and appropriate use of personal data; maintain the trust of participants joining research studies; and to support and enhance compliance with the increasingly robust frameworks that underpin the governance and ethico-legal oversight of the management, use and presentation of research data.

## Data Availability

The process of generating the synthetic data used for the graphical illustrations is described in details in Sect. 3.
